# Dynamic Compression Induced Solidification

**DOI:** 10.3390/polym12020488

**Published:** 2020-02-22

**Authors:** Benedikt Roth, Wolfgang Wildner, Dietmar Drummer

**Affiliations:** Institute of polymer technology, Friedrich-Alexander-Universität Erlangen-Nürnberg (FAU), Am Weichselgarten 9, 91058 Erlangen, Germany; wolfgang.wildner@fau.de (W.W.); dietmar.drummer@fau.de (D.D.)

**Keywords:** no-flow pressure, solidification, polycarbonate

## Abstract

This study presents a method for the determination of the dynamic pressure-dependent solidification of polycarbonate (PC) during flow using high pressure capillary rheometer (HPC) measurements. In addition, the pressure-dependent solidification was determined by isothermal pressure-volume-temperature (pvT) measurements under static conditions without shear. Independent of the compression velocity, a linear increase of the solidification pressure with temperature could be determined. Furthermore, the results indicate that the relaxation time at a constant temperature and compression rate can increase to such an extent that the material can no longer follow within the time scale specified by the compression rate. Consequently, the flow through the capillary stops at a specific pressure, with higher compression rates resulting in lower solidification pressures. Consequently, in regard to HPC measurements, it could be shown that the evaluation of the pressure via a pressure hole can lead to measurement errors in the limit range. Since the filling process in injection molding usually takes place under such transient conditions, the results are likely to be relevant for modelling the flow processes of thin-walled and microstructures with high aspect ratios.

## 1. Introduction

In the course of digitization and electrification, there is an increasing demand for components whose property profile can be adapted over a wide range to the respective application, while at the same time being manufactured economically in large quantities [1]. Two main research areas here are thin-wall and microtechnology as well as the manufacturing of thick-walled optical components in the fields of information and communications technology and the automotive sector [2]. Due to the economical mass production by injection molding and the possibility of function integration as well as the low specific weight, polymers feature significant advantages over metals or glasses in these areas of application [3,4].

Therefore the demand for injection-molded components, which have to meet ever higher requirements, is constantly increasing in the microtechnology and optics sectors, with the often difficult processing of such components requiring intensive research work [5,6]. In order to reduce the iteration effort in the design of such manufacturing processes, advanced computer simulations are often used today. By knowing the material’s behavior under process-related conditions, it is possible to determine the limits of the process in relation to complex component geometries in iterative simulations even before the molds are manufactured, thus avoiding cost-intensive rework loops. However, one parameter still often neglected in filling and cooling simulations is the influence of pressure on the flow and solidification behavior of the polymer melt.

At a constant pressure, an amorphous polymer has a defined free volume at any temperature at which the molecular chains are in the most favorable energetic state [7,8,9]. This state changes with a temperature or pressure variation [10,11,12]. Due to the viscoelasticity of the polymers, however, they can only follow a change in pressure or temperature as a function of time in order to reach the new state [13]. This time is determined by the mobility of the molecular chains within the available free volume and is therefore also pressure and temperature dependent [14,15]. The mechanism of the glass transition is thus determined by the considered time scale, within which a new state results, and the relaxation time, required to complete the structural changes, so that this state is reached [16]. Previous investigations on the pressure dependence of the glass transition are limited to specifying the time scale under consideration by a constant cooling rate in isobaric pressure-volume-temperature (pvT) measurements or a constant pressure increase in isothermal pvT measurements [17]. During these measurements, the relaxation time is determined by a static pressure and the temperature decrease or by a static temperature and the pressure increase, respectively. If the relaxation time is shorter than the considered time scale, the polymer is in a molten state. However, if the relaxation time exceeds the considered time scale as cooling progresses, the polymer falls out of equilibrium and solidifies [11,18]. This leads to the known dependence of the glass transition on the static pressure or the cooling rate. At a constant cooling rate, a higher pressure leads to an increased relaxation time due to the limited mobility of the polymer chains [19,20,21]. At constant pressure, a higher cooling rate leads to a reduction of the considered time scale and therefore to a shift of the glass transition to higher temperatures [22]. Conversely, the considered time scale can also be determined by the velocity of isothermal compression [23]. The relaxation time is then determined by the restriction of molecular mobility due to the existing pressure level [24,25]. For Example the pressure dependence of the glass transition at a constant cooling rate of 3 K/min is 0.12 K/MPa for polymethylmethacrylate (PMMA) and 0.3 K/MPa for polycarbonate (PC) [17].

**Figure 1:**
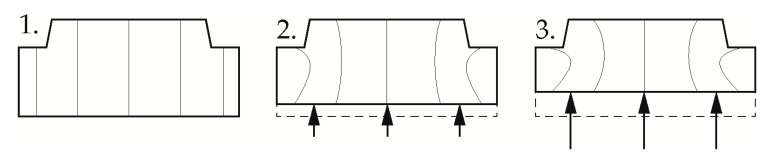
Melt flow during compression in pressure-induced solidification (CIS); **1**: No Compression; **2**: Beginning compression; **3**: End of Compression; black arrows indicates the compression direction.

Current research at the Institute of Polymer Technology focuses on the use of the pressure dependence of the solidification behavior in the injection molding process of pressure-induced solidification (CIS) for the production of thick-walled optical components. Here, special challenges arise with regard to an economic cycle time with simultaneously good optical properties and high dimensional accuracy [26]. Due to the poor thermal conductivity of the polymer in combination with the large wall thicknesses, cooling times of up to 20 min result in conventional production of such components by injection molding [27]. In addition, the two-phase cooling of the components often results in residual stresses, sink marks and distortion. These represent rejection criterions, especially in the field of imaging optics [26]. In CIS, the application of a high pressure at a temperature close to the glass transition decouples the solidification from the cooling, with the shrinkage of the component in the subsequent cooling step taking place over the entire cross-section with the same coefficient of thermal expansion. This results in components, free of residual stresses and with high dimensional accuracy [28]. In this processing technique, the melt is injected into the cavity, which has a temperature above the glass transition. After a so-called adaptation time, during which the polymer cools down to the mold temperature, the melt is compressed by a piston. However, the one-sided compression movement results in local pressure differences between thinner and thicker component areas, which in turn induces flow processes to the center of the component [29]. Figure 1 shows the expected melt flow within the cavity cross-section with one-sided compression as a temporal sequence.

A second example in which unintentionally high-pressure gradients can occur, is micro injection molding. In the production of thin-walled structures in injection molding, flow path and cross-section-dependent pressure and temperature differences can often lead to difficult mold filling, especially during the injection phase [30]. Previous approaches have focused on reducing the viscosity of the melt through high melt temperatures or dynamic mold temperature control to such an extent that the cavity can still be completely filled [31,32]. Another approach to reducing viscosity, by shearing while simultaneously avoiding extended cycle times due to higher mold or melt temperatures, is to increase the injection velocity. Due to the high aspect ratios in micro injection molding, this results in injection pressures of up to 3000 bar if the injection velocity is too high [33].

The solidification behavior of polymer melts at the glass transition as a function of temperature and time has already been investigated in terms of free volume using positron annihilation lifetime spectroscopy [10,15] and conventional pvT-measurements [8]. There have also been investigations on the relaxation behavior above and below the glass transition using stress relaxation experiments [34], and a combination of dynamic dielectric spectroscopy and thermostimulated depolarization currents [35]. However, the steady-state conditions which have always been the basis of previous investigations on the dependencies of glass transition do not reflect the real process either in micro injection molding or in CIS, since the material is not influenced statically but dynamically by pressure and temperature gradients. Several investigations on the pressure dependence of viscosity have shown [36,37,38,39,40,41,42,43,44,45,46,47] that the free volume and thus also the relaxation time are influenced by pressure. Therefore, a solidification due to a temporal pressure gradient at a static temperature would also be conceivable, according to the model of solidification explained above. In this case the compression velocity determines the considered time scale. An indication of such a dynamic pressure-dependent solidification can be found in [48]. By combining the free volume Williams-Landel-Ferry (WLF) equation to describe the zero viscosity with the Bueche and Graessley equation [49] to describe the shear-thinning material behavior and the momentum equation in the capillary flow, the axial pressure distribution in the capillary could be calculated. For PC, at a temperature of 250 °C and a shear rate of 300 s^−1^, it was determined that at high length to diameter (L/D) ratios of the capillary the pressure loss across the capillary shows a nonlinear course. The representation of the capillary pressure as a function of the L/D ratio of the capillary also shows a nonlinear behavior of the capillary pressure for high L/D ratios and high shear rates, which indicates a solidification of the material at the pressure sensor or at the capillary inlet. [48] This effect was also registered by [50] for PMMA. A proof of the dynamic pressure-dependent solidification can be found in [51]. Capillary rheometer measurements at different temperatures on polyethylene showed an exponential increase in viscosity at the lowest melt temperature of 130 °C from approx. 1000 bar pressure. This was attributed to an incipient crystallization at the capillary inlet. The collapse in viscosity from approx. 1800 bar onwards was attributed to an increasing solidification of the material at the capillary inlet and thus, prevented the formation of a flow profile [51].

Up until now, the solidification behavior of amorphous polymers due to a pressure change during flow has hardly been investigated. Particularly in the application cases of micro injection molding and CIS, knowledge of the temporal pressure gradient at which the polymer flow in the mold can stop (no flow pressure) is indispensable for a simulative determination of the process limits. Therefore, the aim of this investigation is to determine the pressure-dependent solidification of the melt as a function of temperature, compression velocity, and shear. Isothermal pvT measurements were performed to determine the pressure-dependent solidification without shear under static conditions. To determine the pressure-dependent solidification during flow, high pressure capillary rheometer (HPC) measurements were evaluated with a new method. These measurements were carried out in overlapping temperature ranges to check the calculation of a master curve for implementation in existing simulation models.

## 2. Materials and Methods

### 2.1. Materials

Due to the already described high pressure dependence of the glass transition of 0.3 K/MPa, a PC, type Makrolon LQ2647 (Covestro AG, Leverkusen, Germany), with a glass transition temperature of 144 °C [52] was used for the analysis.

### 2.2. Methods

The isothermal pvT measurements were performed at five temperatures of 170 °C, 185 °C, 195 °C, 205 °C and 220 °C and in a pressure range from 500 bar to 2500 bar with a pressure increment of ∆*P* = 100 bar. A Rheograph 25 (Goettfert Werkstoff Prüfmaschinen GmbH, Buchen, Germany) with a piston diameter of 9 mm was used for these measurements. The maximum force of the device is 16 kN, which corresponds to a pressure of 2500 bar. After each increase of pressure, the unit was held for 2 min to ensure steady state conditions. Table 1 shows an overview of the conducted pvT experiments.

**Figure 2:**
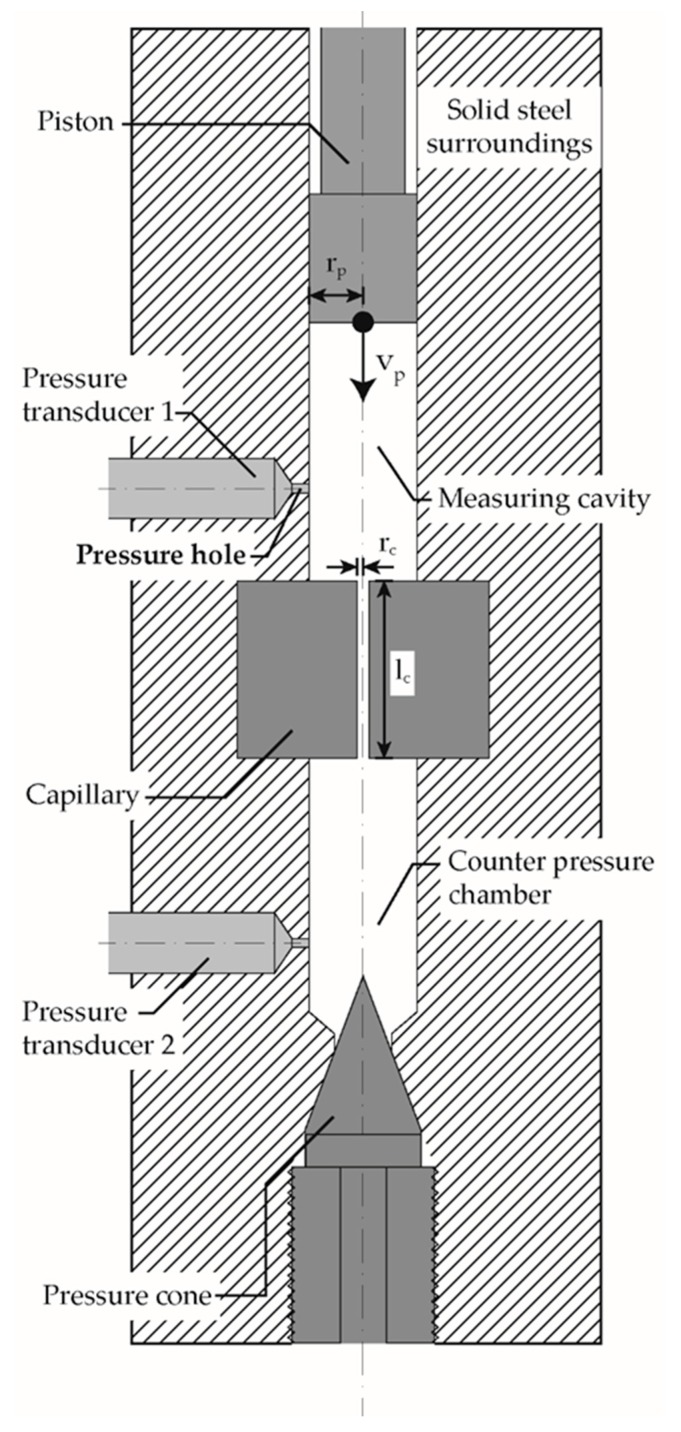
Schematic drawing of the counter pressure Rheograph 75 with the pressure sensor behind the pressure hole; adapted according to [53]; *r*_p_: piston radius; *v*_P_: piston velocity; *r*_c_: capillary radius; *l_c_*: capillary length.

The analysis of the pressure-induced solidification during flow was carried out with a counterpressure Rheograph 75 (Goettfert Werkstoff Prüfmaschinen GmbH, Buchen, Germany) with a piston diameter of 15 mm. The measuring device used was specified with an accuracy of 0.4% for pressures of 20–2500 bar, whereby the maximum pressure corresponds to a force of 40 kN. To increase accuracy, the elasticity and deformation of the frame, the drive train and the force transducer are calculated as a function of the stamp force and automatically corrected. Figure 2 shows the test setup schematically based on [53]. The measuring chamber was first heated to the test temperatures of 220, 230, 240 and 250 °C, and then filled in several steps with polymer granulate, manually compressed and degassed until the chamber was completely filled without air inclusions. In order to ensure the melting of the polymer, the piston was lowered onto the melt at a pressure of 2 MPa and held at the respective temperature for 5 min. Afterwards the molten polymer charge was pressed through an installed capillary and the counterpressure chamber. After the entire system was filled with degassed polymer melt, the counterpressure chamber was closed by screwing in the pressure cone, Figure 2. The temperature was maintained for a further 5 min, followed by compression. During compression at different piston velocities (0.0028 mm/s, 0.056 mm/s, 0.028 mm/s) the following data were recorded at a frequency of 2 Hz: piston position and velocity, piston force and pressure at the pressure sensors type HDA 2174 (Goettfert Werkstoff Prüfmaschinen GmbH, Buchen, Germany) in the measuring cavity p1 and in the counterpressure chamber p2. The piston force was converted into the piston pressure p_p_ with the chambers cross-section of 176.71 mm^2^. The volume of the measuring cavity was 28 cm^3^, the volume of the counterpressure chamber was 2.73 cm^3^, the capillary’s length to diameter ratio was 20/1. Table 2 shows an overview of the conducted HPC experiments.

### 2.3. Measurement Evaluation

**Figure 3:**
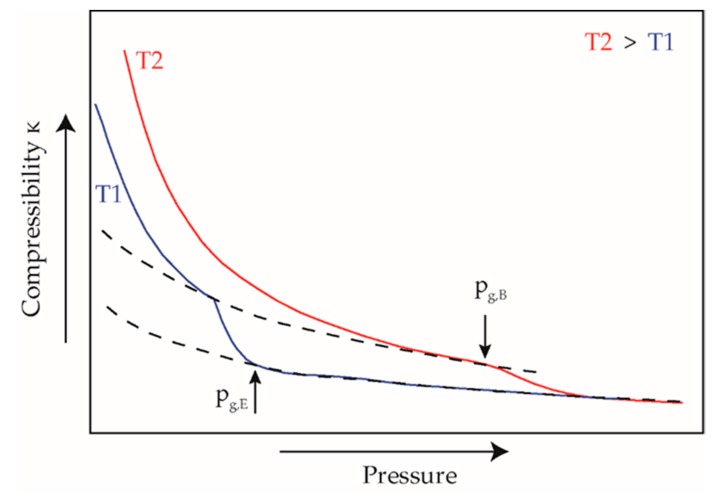
Calculated isothermal compressibility for evaluation of solidification pressure in pvT measurements, adapted according to [54]; *p*_g,E_: pressure of ending solidification; *p*_g,B_: pressure of beginning solidification.

The evaluation of the solidification under static conditions in the isothermal pvT measurements was based on [54,55] by determining the compressibility. If the specific volume is plotted as a function of the pressure at the measured isothermal temperatures, the slope of the curves corresponds to the compressibility. According to [54], the specific isothermal compressibility can be obtained by calculating the first derivative of the specific volume with respect to the pressure:

(1) $$\kappa =-\frac{\delta v}{\delta p}$$

where *κ* is the isothermal compressibility, *v* is the specific volume, *p* is the pressure. The gradient change in the curve of the specific compressibility indicates a change in compressibility to lower values due to the solidification of the material at the glass transition. After solidification, the compressibility remains at the constantly low level of a solid, Figure 3.

By calculating the second derivative of the specific volume with respect to the pressure, the turning point of the compressibility, as well as the beginning and end of the slope change, can be evaluated. The result is the pressure-dependent glass transition range for the measured isothermal temperatures.

**Figure 4:**
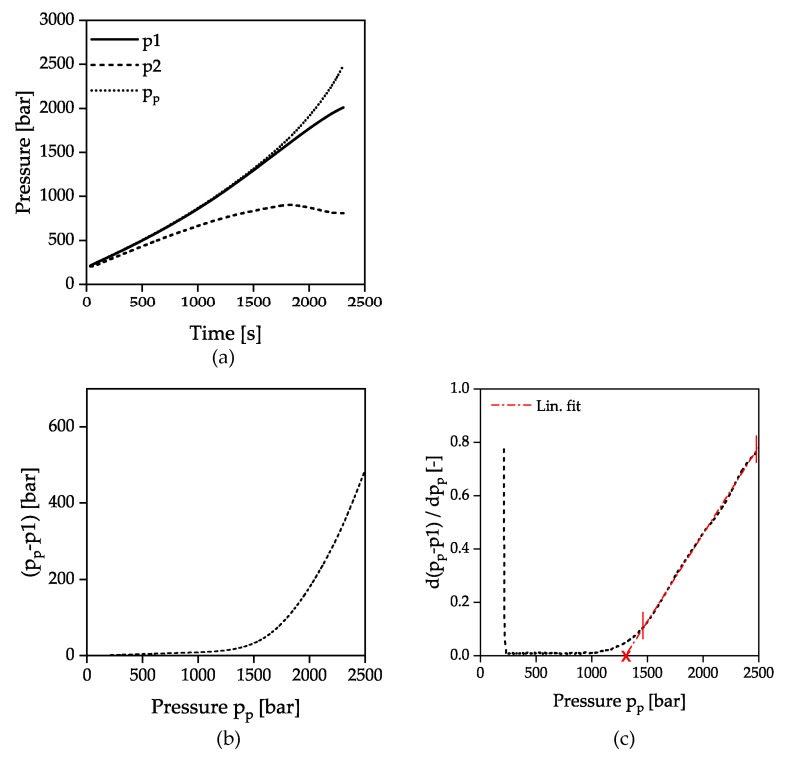
Determination of the solidification by evaluation of the pressure difference in HPC measurements; p1: Pressure transducer measuring cavity p2: Pressure transducer counter pressure chamber p_p_: Pressure calculated from piston force; (**a**) pressure signals as a function of time; (**b**) pressure difference p_p_-p1 as a function of p_p_; (**c**) first derivation of (**b**) with respect to p_p_.

The evaluation of the solidification of the material during flow under dynamic conditions (HPC measurements) was carried out as described in Figure 4. Considering the course over time of both pressure sensors p1 and p2, as well as the pressure p_p_ calculated from the piston force in Figure 4a, it can be seen that initially all pressures increase linearly due to the constant piston velocity and the compression of the material in the measurement and the counter pressure chamber. The resulting pressure difference between the sensors p1 and p2 is due to the pressure loss caused by capillary flow as well as to the inlet and outlet pressure loss.

At a pressure of approx. 1000 bar, the pressure p1 and the piston pressure p_p_ begin to diverge. This was attributed to the fact that the pressure can no longer be transferred correctly through the pressure bore to the pressure sensor due to the incipient solidification at the capillary inlet. At the same time the stopping flow through the capillary prevents further pressure transmittance to the pressure sensor p2 and the slope decreases. The pressure at the time of the divergence between the signals p1 and p_p_ was defined as the solidification criterion. The representation of the pressure difference Δ*p* between p_p_ and p1 as a function of p_p_ shows a parabolic curve with nearly linear increasing gradient after reaching a certain pressure, Figure 4b. The vertex of the parabola marks the solidification and was determined by the intersection of a linear fit in the high-pressure range with the x-axis applied to the first derivative of the pressure difference with respect to p_p_. The intersection point with the x-axis is marked by a cross, as shown in Figure 4c. It should be noted that the linear extrapolation to the point of intersection with the x-axis does not mark the exact beginning of the solidification, since the difference from the pressure signals seems to increase already from approx. 1000 bar. The inconstant gradient of the pressure signal p_p_ after diverging indicates that the glass transition is completely passed through. Due to the force limitation of the apparatus at 2500 bar, no clear limitation of the solidification range similar to the isothermal pvT measurements can be made.

## 3. Results and Discussion

**Figure 5:**
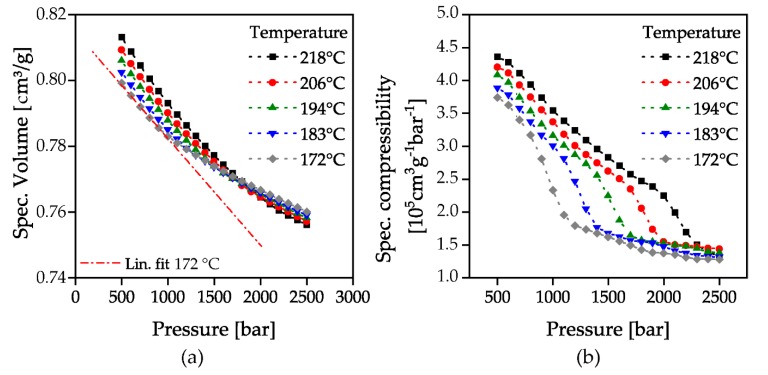
Isothermal pvT-measurements (**a**) and calculated specific compressibility according to Equation (1) (**b**).

**Figure 6:**
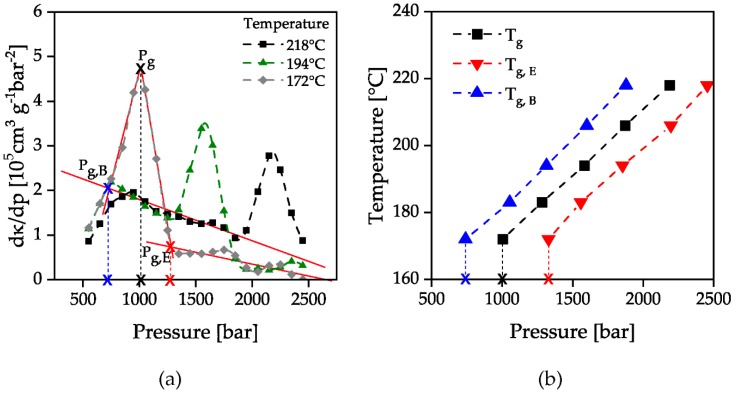
Second derivative of the specific volume (**a**) and evaluation of the temperature dependent glass transition (**b**); *p*_g,B_: pressure of beginning solidification; *p*_g,E_: pressure of ending solidification; *T*_g,B_: temperature of beginning solidification; *T*_g;E_: temperature of ending solidification.

Figure 5 and Figure 6 show the results of the determined pressure-dependent solidification at the investigated temperatures without flow in isothermal pvT measurements to. The specific volume of all temperatures decreases with increasing pressure. The higher the temperature, the greater the decrease, since the compressibility of the material is higher consistently with the higher mobility of the polymer chains. It is also interesting to note that at a pressure of about 1800 bar all investigated temperatures have the same specific volume and with further pressure increase the effect reverses. With classical plotting in the pvT diagram, this is equivalent to a volume decrease with decreasing temperature at low pressures (negative slope of isobars below the glass transition), a volume constancy with cooling at a pressure of 1800 bar (slope = 0) and a volume expansion with cooling at a pressure >1800 bar (positive slope of isobars below the glass transition). The hypothesis for this is, that the pressure stored in the material counteracts shrinkage due to the decrease in temperature by volume expansion. This can be used in a targeted manner in the context of an isochoric compression injection molding process to compensate the uneven shrinkage of thick-walled components. This is the subject of current investigations at the institute of polymer technology.

As described above, the specific volume of the material exhibits a slope change at a certain pressure, Figure 5a. To illustrate this, a linear fit for the temperature of 172 °C in the low-pressure range was added. This marks the passing of the glass transition by the corresponding pressure. The specific compressibility calculated via Equation (1) from the specific volume curves is shown in Figure 5b. At each temperature, the compressibility decreases almost constantly with increasing pressure, until the slope change indicates the beginning of the pressure-induced glass transition. At the end of this glass transition, the compressibility remains at an almost constant level.

**Figure 7:**
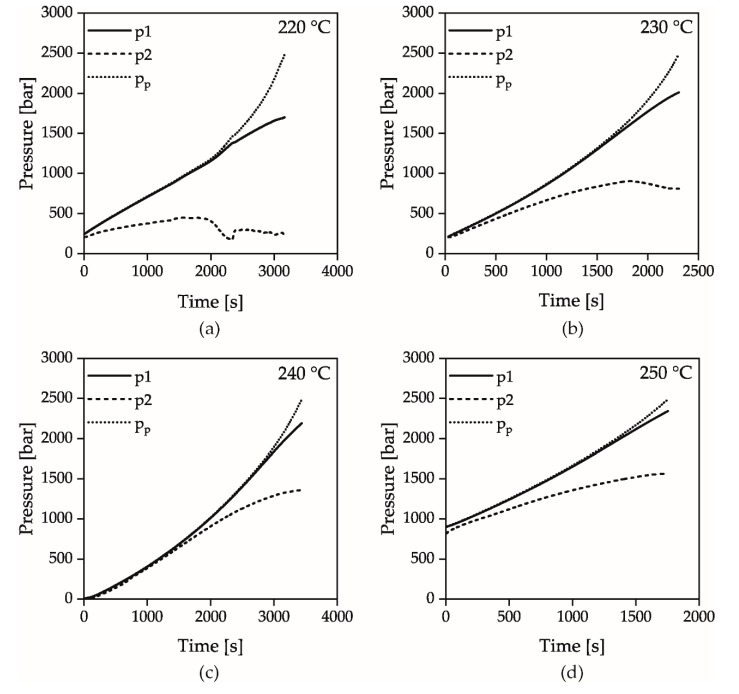
Pressures p1, p2 and p_p_ for the Temperatures 220 (**a**), 230 (**b**), 240 (**c**) and 250 °C (**d**); the velocity of the piston was 0.0056 mm/s.

The beginning and end of the glass transition was determined by the attachment of tangents to the parabolic curves of the first derivative of specific compressibility, Figure 6a. The curves of specific compressibility (Figure 5b) show a change in curvature as the glass transition is passed through. When the solidification pressure is reached, the curves show a right-hand curvature which then changes to a left-hand curvature at the turning point. This is illustrated by the first derivative of the specific compressibility (Figure 6a), which shows a maximum at the point of maximum curvature change. The areas with the same curvature correspond to the compressibility before reaching or after passing through the glass transition. This is represented by the constant slope of the first derivative of the specific compressibility before and after the maximum in Figure 6a. By the intersection of the attached tangents of the second derivative’s maximum with the constant slopes before and after passing through the glass transition, the beginning and the end of the glass transition can be evaluated. The evaluated points are marked with a red-colored cross in Figure 6 as an example for the temperature 172 °C. The evaluated solidification pressures were then plotted over the investigated temperatures. The resulting number of the beginning and end of the glass transition is shown in Figure 7b together with the pressure with a maximum change in compressibility. A linear increase of the solidification pressure with increasing temperature could be proven. The increase of the glass transition is approx. 25 bar/K and is therefore lower than in previous investigations (33–37 bar/K) [17]. The reason for this could be that these measurements were carried out in a stationary state with a waiting time after each pressure increase instead of a constant compression.

Figure 7 shows the pressures p1, p2 measured via the pressure transducers and the pressure p_p_, which was calculated from the piston force, at the temperatures 220, 230, 240 and 250 °C when the counter pressure chamber is closed and the piston velocity is 0.0056 mm/s. As visible, the pressure at which the signals p1 and p_p_ begin to diverge increases with increasing temperature. This trend could be shown in all experiments independently of the piston velocity and reflects the measured solidification behavior of the isothermal pvT measurements. At higher temperatures, the increased mobility of the polymer chains must therefore be compensated by higher pressures in order to solidify the material according to the described mechanism. At a pressure p_p_ of 2500 bar, the measuring device stops the piston because its maximum force has been reached.

**Figure 8:**
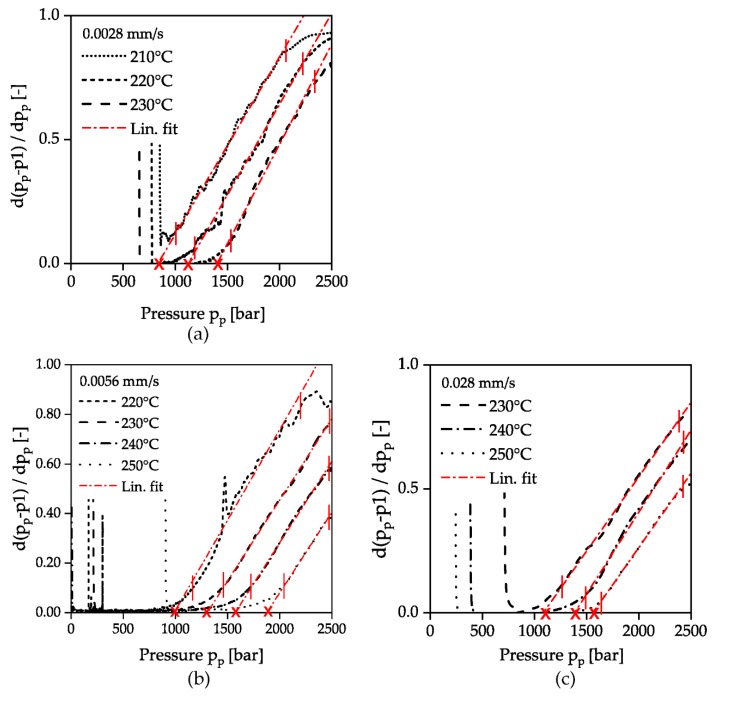
Evaluation of the beginning of solidification at different compression velocities. The ordinates of the graphs show the first derivative of the difference of the pressure signals p1 and p_p_. The vertical red lines indicate the range for building the linear fit. The crosses mark the intersections with the x-axis for the different temperatures; (**a**): 0.0028 mm/s; (**b**): 0.0056 mm/s; (**c**): 0.028 mm/s.

As described in Section 2.3 (Figure 4), the evaluation was carried out according to the specified solidification criterion by plotting the first derivative of the deviation between the pressure signals p_p_ and p1 as a function of the pressure p_p_. The evaluation of all investigated temperatures and compression velocities are summarized in Figure 8. After an area, with no difference between the signals p1 and p_p_, the deviation increases exponentially when the solidification pressure is reached. In this range, the calculated derivative of the difference between the pressure signals p1 and p_p_ in Figure 8 shows a linear course in all experiments performed, which is why a solidification pressure could be determined by linear extrapolation. The intersection of each linear fit with the x-axis is marked by a cross. As it can be seen in Figure 8a higher piston velocity results in a shift of the incipient solidification to lower pressures p_p_. This reinforces the assumption that solidification due to pressure time derivatives can already occur at lower pressures. According to the mechanism described, the considered time scale decreases with higher compression velocity, while the relaxation time increases at the same time due to the increase in pressure and the specified constant temperature. The result is that even at lower pressures the polymer can no longer follow the externally applied changes and thus falls out of equilibrium and solidifies.

**Figure 9:**
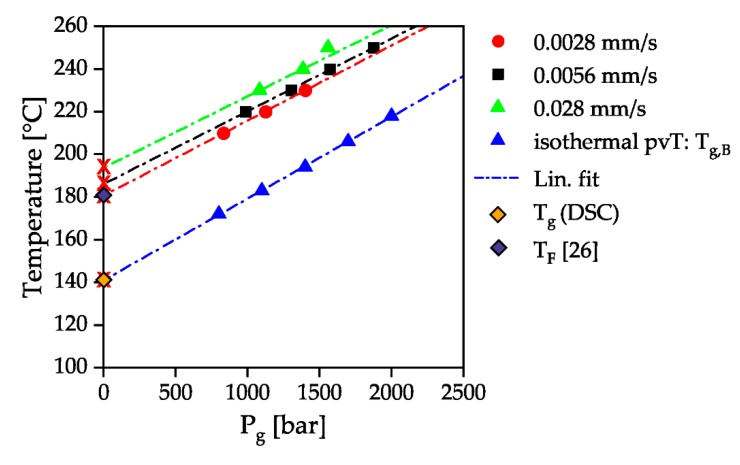
Pressure of solidification *P*_g_ in relation to the temperature for the four different measurements: isothermal pvT-measurement, dynamic solidification with a closed counter pressure chamber and a piston velocity of 0.0028 mm/s, 0.0056 mm/s and 0.028 mm/s.

Figure 9 summarizes the beginning of pressure-induced solidification for the measured temperatures and compression velocities of various measurements. A linear adjustment was made for the isothermal pvT measurements and for the HPC measurements with the counter pressure chamber. The inclination of the HPC measurements is 28.40 bar/K at a piston velocity of 0.0028 mm/s, 29.33 bar/K at a piston velocity of 0.0056 mm/s and 29.9 bar/K at a piston velocity of 0.028 mm/s. The temperature dependency of the pressure-dependent solidification of the material is thus independent of the compression velocity and is in good agreement with the gradients of 37 and 33 bar/K determined by Rudolph et al. [17] for different types of PC. A line with an average gradient of 26 bar/K was added to the measured beginning solidification curve of the isothermal pvT measurement. It can be seen that this linear fit intersects the y-axis at a temperature of 142 °C, which is close to the glass transition temperature of 144 °C measured by DSC according to ISO 11357 at a heating and cooling rate of 10 K/min without pressure influence. The intersection of the pressure-dependent glass transition with the x-axis is 180 °C for a piston velocity of 0.0028 mm/s. This is in good agreement with the determination of the no-flow temperature by the intersection of storage and loss module at a low frequency of 0.1 Hz in a dynamic mechanical analysis for the same material type of [29]. The temperature dependence of the solidification pressure is therefore independent of the flow of the melt. When flowing through a capillary with a constant piston velocity, however, solidification can occur at relevant processing pressures (1500 bar, 250 °C). At a higher piston velocity, solidification at the processing temperature of 280 °C, recommended for PC in the material data sheet, would therefore also be conceivable.

The results show that the pressure of solidification varies with both temperature and deformation rate. A higher deformation rate results in the pressure-induced solidification starting at lower pressures. This behavior confirms the theory of solidification of the material during flow due to excessive temporal pressure gradients. The stop of the flow through the capillary is confirmed by the diverging course of the piston pressure p_p_ and the pressure sensor of the measuring chamber from the beginning of solidification or by the decrease in pressure at the pressure sensor in the counterpressure chamber. One consequence that should be drawn from the measurement results is that the evaluation of the pressure via a pressure bore in HPC measurements in the limit range can lead to measurement errors. Furthermore, the results indicate that the material can solidify under process-related temperatures and temporal pressure gradients. Therefore, consideration of this behavior is necessary to improve the prediction accuracy of simulation models, especially for optical applications and in the field of thin wall and micro technology.

**Figure 10:**
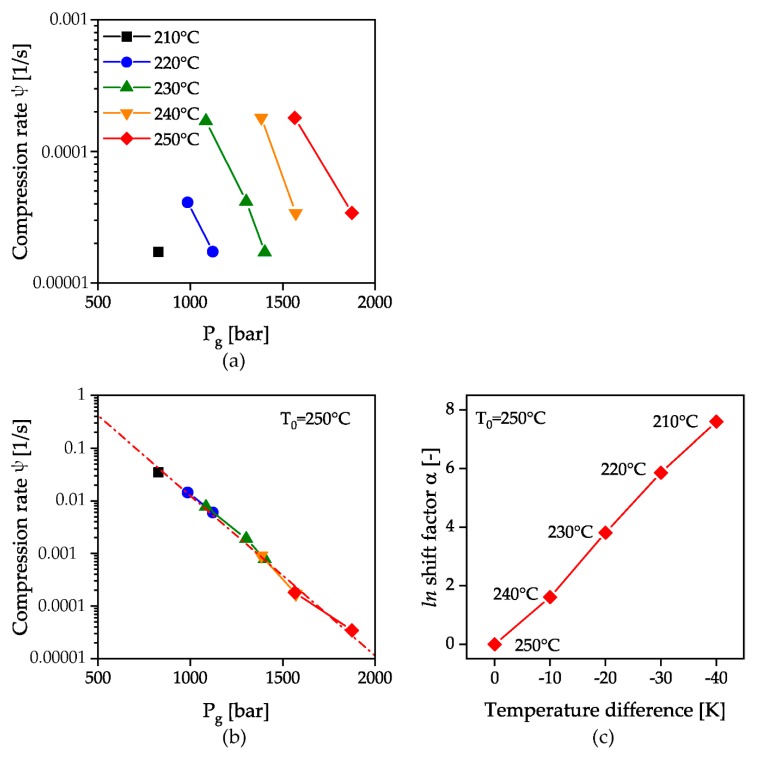
HPC measurements as a function of a measurement instrument independent compression rate ψ (**a**); calculation of a master curve by shifting the measurements with a shift factor α (**b**); logarithmic shift factor α as a function of the temperature difference to the reference temperature of 250 °C (**c**).

If the rate of change of the volume dV/dt is calculated from the product of the piston velocity and the piston area and this is related to the total chamber volume V, the compression rate

(2) $$\psi =\frac{1}{V}\frac{dV}{dt}$$

is obtained. This is independent of the geometries and setting parameters of the measuring instrument. The logarithmic representation of the solidification pressure from the HPC measurements for all measured temperatures as a function of this calculated compression rate is shown in Figure 10a. A constant increase of the solidification pressure with temperature can be observed with simultaneous linear decrease of the solidification pressure with exponentially increasing compression rate. By shifting the temperature curves at a reference temperature of 250 °C as a function of the compression rate with a shift factor α, the temperature invariant representation of a master curve to describe the solidification behavior during flow appears possible, Figure 10b. The shifting coefficients were calculated by dividing the compression rate at the desired temperature by the compression rate of the reference temperature, and are shown in Figure 10c.

An exponential fit of the shifted temperature curves yields an equation for describing the master curve at 250 °C:

(3) $$\psi_{250\;{}^{\circ}C}=\exp\left(2.62-0.007\times p_{g}\right)$$

wherein the Pearson correlation coefficient is 0.99. The displacement of this curve as a function of temperature is taken into account by the temperature displacement factor α and the corresponding temperature difference to the reference temperature Δ*T*:

(4) $$\psi_{T}=\exp\left(2.62-0.007\times p_{g}+\Delta T\times \alpha \right)$$

This relationship is valid for the material investigated. The transferability of the results to other materials is the subject of current investigations at the Institute of polymer technology.

## 4. Conclusions

The results on the dynamic solidification of PC and its comparison with static isothermal pvT measurements have shown that the beginning of the solidification process depends on temperature, pressure and compression rate. In addition to the static representation of the solidification by a change of the specific compressibility, a more process-oriented dynamical representation by means of HPC measurements seems possible. In both measuring methods, a linear increase of the solidification pressure with temperature could be recorded. In addition, it could be shown that a higher compression rate leads to solidification at lower pressures, analogous to solidification at higher temperatures by higher cooling rates. The results indicate that the evaluation of the pressure via a pressure hole in HPC measurements in the limit range can lead to measurement errors. The results of the calculation of the master curve indicate that solidification can be possible at temperatures relevant to processing due to excessive pressure gradients. After further research, this material behavior should therefore be implemented in computer simulations to improve the prediction accuracy. The presentation of the results of the HPC measurements as a master curve appears possible.

## Figures and Tables

**Table 1:** Overview of the pvT experiments.

Temperature [°C]	Pressure Range [bar]	Pressure Increment Δ*p* [bar]
172 °C	500–2500	100
183 °C	500–2500	100
194 °C	500–2500	100
206 °C	500–2500	100
218 °C	500–2500	100

**Table 2:** Overview of the high pressure capillary rheometer (HPC) experiments.

Piston Velocity [mm/s]	Temperature [°C]
0.0028	210
	220
	230
0.0056	220
	230
	240
	250
0.028	230
	240
	250
